# Evidence for microvesicle particles and platelet-activating factor as effectors for systemic effects of thermal burn injury

**DOI:** 10.3389/fimmu.2026.1771102

**Published:** 2026-02-24

**Authors:** Akhil Varghese Parackal, Richard C. Fox, Aadil Umerani, Youngjun Park, R. Michael Johnson, Wenfeng Zhang, Zheng Xu, Alison A. Smith, Craig A. Rohan, Jeffrey B. Travers

**Affiliations:** 1 Department of Pharmacology and Toxicology, Boonshoft School of Medicine at Wright State University, Dayton, OH, United States; 2 Department of Plastic Surgery, Boonshoft School of Medicine at Wright State University, Dayton, OH, United States; 3 Department of Mathematics and Statistics, Wright State University, Dayton, OH, United States; 4 Department of Surgery, Louisiana State University Health Sciences Center, New Orleans, LA, United States; 5 Department of Medicine, Dayton Veterans Administration Medical Center, Dayton, OH, United States; 6 Department of Dermatology, Boonshoft School of Medicine at Wright State University, Dayton, OH, United States

**Keywords:** alcohol, microvesicles, multiple organ dysfunction, platelet-activating factor, thermal burn injury

## Abstract

Thermal burn injury (TBI) is an important source of morbidity and mortality. The exact mechanisms for the systemic effects including multiple organ dysfunction (MOD) and immune deficits associated with extensive skin burn injuries are unclear and this knowledge gap has negatively impacted therapy. The goal of this review is to present evidence for a model of TBI-induced systemic effects that involves skin keratinocyte release of subcellular microvesicle particles (MVP) carrying the potent lipid mediator Platelet-activating Factor (PAF) as effectors. As TBI has been shown to generate high levels of potent 1-alkyl PAF versus lesser amounts of inhibitory 1-acyl PAF species, it would be expected that MVP produced by burn would be highly powerful PAF receptor agonists. In addition, we present results of a pilot study with 12 human subjects suggesting that MVP can be measured systemically within hours following a TBI. This model involving MVP provides an explanation for why advanced age and ethanol intoxication result in worsening systemic effects including MOD following a TBI. The need for more clinical studies in this critical area of TBI pathogenesis is also discussed. Potential therapeutic implications of this model will also be addressed. An improved understanding of how a significant skin burn injury results in systemic manifestations will result in improved TBI patient outcomes and could be applicable to other environmental stressors.

## 1 Introduction

According to the World Health Organization, burns are defined as cellular and tissue injuries primarily caused by heat, radiation, radioactivity, electricity, and the toxic effects of applied chemicals. These injuries drive approximately 400,000 individuals in the United States every year to seek medical treatment, and worldwide it is estimated that burns cause at least 265,000 mortalities annually (1). These injuries incur over 300,000 US-based inpatient hospitalizations and an estimated 40% of those injuries occur from flames and other thermal processes (2). Thermal burn injuries, or TBI’s, can present an acutely urgent issue with typical treatment sought either within minutes to hours for severe incapacitating burns to within the first two days following lesser injuries. The time before seeking medical attention is crucial to improve the mortality of an individual pending the extent of the burn (3).

The prevalence of burns worldwide exceeds an estimated six million injuries yearly with over 265,000 of those individuals dying from the effects of the burns (1, 4). Care of the severe TBI victim imposes a yearly cost of hundreds of thousands with financial burdens ranging from approximately $270,000 to $361,000 per individual. As a country these costs in the United States amount to over one billion dollars annually (5) with increasingly long hospital stays and increased costs when concomitant inhalation burns and increased body surface areas are affected (6). Contributors to the rising costs of these injuries, including a higher incidence of the injury as well as increasing costs for medical treatment and resources allocated to these patients, have rationalized the presence of multiple dedicated burn centers around the country. These burn centers have resulted in the overall improvement of the both acute and chronic care for the TBI patient (7).

## 2 Systemic effects of TBI

### 2.1 Background on TBI systemic effects

Thermal burn injuries, apart from damage to the superficial skin layers, cause systemic pathologies to individuals including but not limited to ischemic necrotic bowel disease (8), acute renal injury (9), bacteremia and associated systemic infectious processes, shock with hypoperfusion of end organs, muscle wasting (10), metabolic consequences, disseminated intravascular coagulation (11) and many more (12). Burns initiate a cascade of cellular processes including coagulation, cytokine induced inflammation, and subsequent tissue granulation at later stages of the pathophysiologic process. These injuries can result in low rates of perfusion following hyperemia, heightened hematologic levels of cellular components from necrotic tissue, and a state of immunosuppression (5).

Following the Jackson model of the burn wound, burns produce three distinct zones related to the damage incurred. The first zone is coagulation where the most tissue damage occurs. Irreversible tissue loss occurs due to the coagulation of abundant proteins within the cellular matrix. The second region is the zone of stasis. In this zone, tissue has decreased perfusion however as a whole can be deemed viable; this zone surrounds the coagulation zone. The third area is the zone of hyperemia. This zone is the most peripheral from the central burn and has the least cellular injury of the three zones. Here also lies the most perfused of the three zones and thus exhibits the highest probability of tissue recovery (13). The actual size of the injury is important as systemic effects occur with higher incidence following an approximately >20% surface area injury (14, 15) As will detail further in this review, systemic effects appear to be in great part due to products of the systemic inflammatory response. The pathologic abnormalities noted following severe TBI include widespread inflammation, increased vascular permeability, inducing disseminated intravascular coagulation, dehydration, hemolysis, and other cellular and tissue death.

### 2.2 Risk factors for systemic effects

Burn injuries, judged in severity by depth and total body surface area covered, affect all age groups. However, those most predisposed to severe effects from burns include those >60 years or less than <2 years of age (14). Other factors associated with a poorer prognosis from TBI include concurrent trauma and smoke inhalation, immunosuppression, and ethanol intoxication (12, 16, 17). Obesity, an abundant comorbidity faced today within the United States, has been correlated with increased incidences of systemic bacteremia, deep vein thrombosis, pulmonary embolism, and increased hospital stays following burn injuries (18).

Previous studies have demonstrated that those engaging in risky behaviors, such as utilizing inebriating substances like alcohol, exhibit an increased incidence of TBI. Other risk factors for TBI include utilizing electronic cigarettes that have been shown to detonate with use (19). Dangerous professions from firefighting to working around hot surfaces such as furnaces or stoves also have higher incidences of TBI (20).

### 2.3 Animal models for investigating systemic effects of thermal burn injuries

Animal models have been valuable tools for unravelling the intricate pathways involved in the systemic responses associated with burn injury. *In vitro* models are currently unable to fully stimulate the complex pathophysiology and clinical features of human burn injuries. Thus, animal models are an indispensable tool for researchers to understand both the post-burn pathophysiology as well as to evaluate innovative therapeutic strategies, enabling research that is ethically infeasible in clinical settings. Primary challenges in burn research with animal models include the lack of an ideal animal model which will replicate all the diverse features of human burn trauma (21). Additionally, it can be difficult to produce uniform burn injuries with consistent depth and characteristics for reliable research (22). Despite these challenges, animal models have played a significant role in elucidating both molecular and cellular characteristics that define human burn injuries (23).

In thermal burn research, rodents (mice and rats), pigs, and rabbits are the most used animal models. Mice are often utilized because of their ease of genetic alteration, cost-effectiveness, quick breeding, and reasonable genetic resemblance to humans (21). Burn inflicted on large animal models like sheep, or swine is also considered sufficient to reproduce many findings including a significant hypermetabolic response (24). Rabbits, although less commonly used for burn research, provide intermediate anatomical similarities in skin structure and thickness, offering additional translational value in specific contexts (25).

Depending on the species and the study issue, different techniques are employed to generate burns in animal models. To simulate the unintentional scald injuries observed in pediatric patients, rat and mice models frequently undergo scald burns, which are created by submerging the skin in hot water (26). With contact burns, consistent partial- or full-thickness burns can be produced by applying hot metal objects at regulated temperatures for predetermined amounts of time (27, 28). In order to investigate the combined consequences of thermal and pulmonary injuries, smoke inhalation models are frequently used in conjunction with flame burns, which simulate fire exposure (21).

### 2.4 Murine models of TBI and intoxicated TBI

Murine models are not only essential tools for understanding and evaluating the complex systemic pathophysiology of TBI, they have use in testing potential therapeutic interventions (21). Mice are extensively used due to their manageability, cost-effectiveness, and well-characterized immune system, in spite of their inherent anatomical and physiological distinctions from human skin, such as thinner epidermis and dermis, dense hair, and primary healing through wound contraction (21, 29, 30). Anesthesia, shaving the dorsal surface, and immersing a predetermined total body surface area (TBSA) into hot water, often at 95-100°C for 7–10 seconds to create full-thickness scald injuries are standard methods of burn induction in mice (21, 26, 31). Other methodologies which appear to generate similar findings involve use of metal blocks heated to ~90°C applied to murine skin (27, 28). Immediate administration of pain relief medication like buprenorphine and resuscitation fluids, such as saline are some post burn care measures employed (26, 27, 32).

The detrimental impact and outcomes of murine burn injury and similarities to the human condition have been demonstrated by the research conducted by Elizabeth J. Kovacs and Mashkoor Choudhry and colleagues. Their studies consistently demonstrate that acute ethanol exposure prior to thermal injury significantly exacerbates immune suppression and systemic inflammation (26, 33). Significant findings include pronounced T-cell dysfunction and increased production of pro-inflammatory cytokines, especially interleukin-6 (IL-6), which is positively correlated with immunosuppression and increased mortality (32, 34, 35). Furthermore, work from these investigators has detailed how the combined insult severely compromises gut barrier function, leading to increased intestinal permeability and heightened bacterial translocation to mesenteric lymph nodes, thereby contributing to widespread systemic sepsis and resultant end organ damage (36, 37). There are several lines of investigation which have linked the increased gut bacterial translocation with the subsequent multiple organ dysfunction. First, the TBI-induced intestinal permeability has been found to be due to gut intestinal activation of myosin light chain kinase (MLCK), and pharmacologic inhibition of this enzyme blocks the systemic organ inflammation following intoxicated TBI (38). Second, mice deficient in toll like receptor 4 (which recognizes gram negative bacterial products) are protected from burn-induced systemic inflammation (39). Aged mice, already exhibiting impaired immune responses, show even greater susceptibility to infections and increased mortality after burns which is linked to increased bacterial translocation from the gut (32, 40). Collectively, the model that has emerged from groundbreaking murine studies has indicated that TBI has the ability to signal internally. Yet, the exact potential effectors linking the skin and lung burn injury have not been elucidated until recently.

## 3 Microvesicle particles & platelet-activating factor as mediators for TBI systemic effects

### 3.1 Microvesicles and platelet-activating factor

Microvesicle particles (MVPs), also called large extracellular vesicles, are membrane-bound vesicles that are discharged from the plasma membrane of different cell types in both healthy and pathological situations. Even though the exact mechanism of MVP release from the plasma membrane is not completely known, the involvement of acid sphingomyelinase (aSMase) in some systems have been identified (41). Microvesicle particles have a diameter of around 100 to 1000 nm whereas smaller exosomes released from inside the cell are ~30–150 nm (42–44). Their involvement in intercellular communication, modulating vital processes in cellular homeostasis, immunological regulation, coagulation, and inflammation, accounts for their biological relevance. It should be noted that the biologic effects of subcellular particles such as MVP are due to their carried contents, to include bioactive agents on the particle surface.

Platelet-activating Factor (PAF; 1-alkyl-2-acetyl glycerophosphocholine) is a potent phospholipid mediator and a family of glycerophosphocholine (GPC)-derived lipid mediators implicated in numerous physiological and pathophysiological processes, including inflammation and apoptosis (45, 46). This novel glycerophosphocholine-based mediator PAF was initially described for its ability to cause platelet aggregation but later recognized as a potent activator of various cell types including monocytes, mast cells and leukocytes. PAF species are generated by both enzymatic processes as well as via ROS-mediated oxidation of membrane glycerophospholipids (47). Intradermal administration of PAF can lead to cutaneous inflammation and is present in various inflammatory skin disorders (48–50). PAF binds to a single G-protein-coupled receptor, the PAF receptor (PAFR) to exerts its biological actions (45). Exogenous PAF given to mice can mimic sepsis (51). Though a highly potent and “dangerous” mediator, PAF is quickly inactivated by acetyl transferases as well as alkaline sphingomyelinase found in cells and serum (52, 53).

As is the case with many powerful bioactive agents such as TNF and IL-1, considerable data suggests that the PAF system also has an endogenous antagonist. Once the structure of PAF was elucidated, it was noted that the 1-alkyl PAF species were at least 100 times more potent than the 1-acyl PAF species that were also generated (50, 54). More recent studies have demonstrated that though both 1-alkyl PAF and 1-acyl PAF attach to the same binding pocket of the PAFR, 1-acyl PAF have a lower dissociation rate. Moreover, 1-acyl PAF binding results in structural instability and does not allow proper G protein signaling (55). Consistent with 1-acyl PAF species serving as an endogenous PAFR antagonist, cellular studies have demonstrated that 1-palmitoyl-2-acetyl-GPC pre-treatment blocks the signaling of subsequent 1-hexadecyl-2-acetyl GPC (50, 56). Providing mice with 1-acyl PAF along with 1-alkyl PAF protects mice from lethal acute effects of the latter (57). Relevant to burn injury, previous studies have demonstrated that HaCaT keratinocytes generate only 1-alkyl PAF in response to an experimental TBI, whereas approximately equal amounts of both species are produced in response to other stimuli such as cold injury or ionophore A23187 (57–59).

### 3.2 Structural characteristics and composition of MVP

Microvesicle particles come in a variety of shapes, most often ellipsoid or spherical. Their stable and bioactive lipid bilayer is mostly taken from the plasma membrane of the parent cell and is enhanced with cholesterol and phospholipids. Phosphatidylserine, tetraspanins (CD9, CD63, and CD81), integrins, and selectins are examples of common surface indicators that help with interactions with recipient cells (60). Like all subcellular particles, MVP express surface or internal markers that can be used to identify the cell of origin. For example, skin keratinocytes have been reported to express the epithelial-selective marker calcium sensing receptor (61). Thus, MVP effects in a variety of biological processes influenced by their markers as well as varied bioactive cargo, which includes proteins (growth factors, cytokines, proteases), lipids (sphingomyelin, ceramides), nucleic acids (mRNA, miRNA), and enzymatic components such acid sphingomyelinase (aSMase) (62).

**Figure 1:**
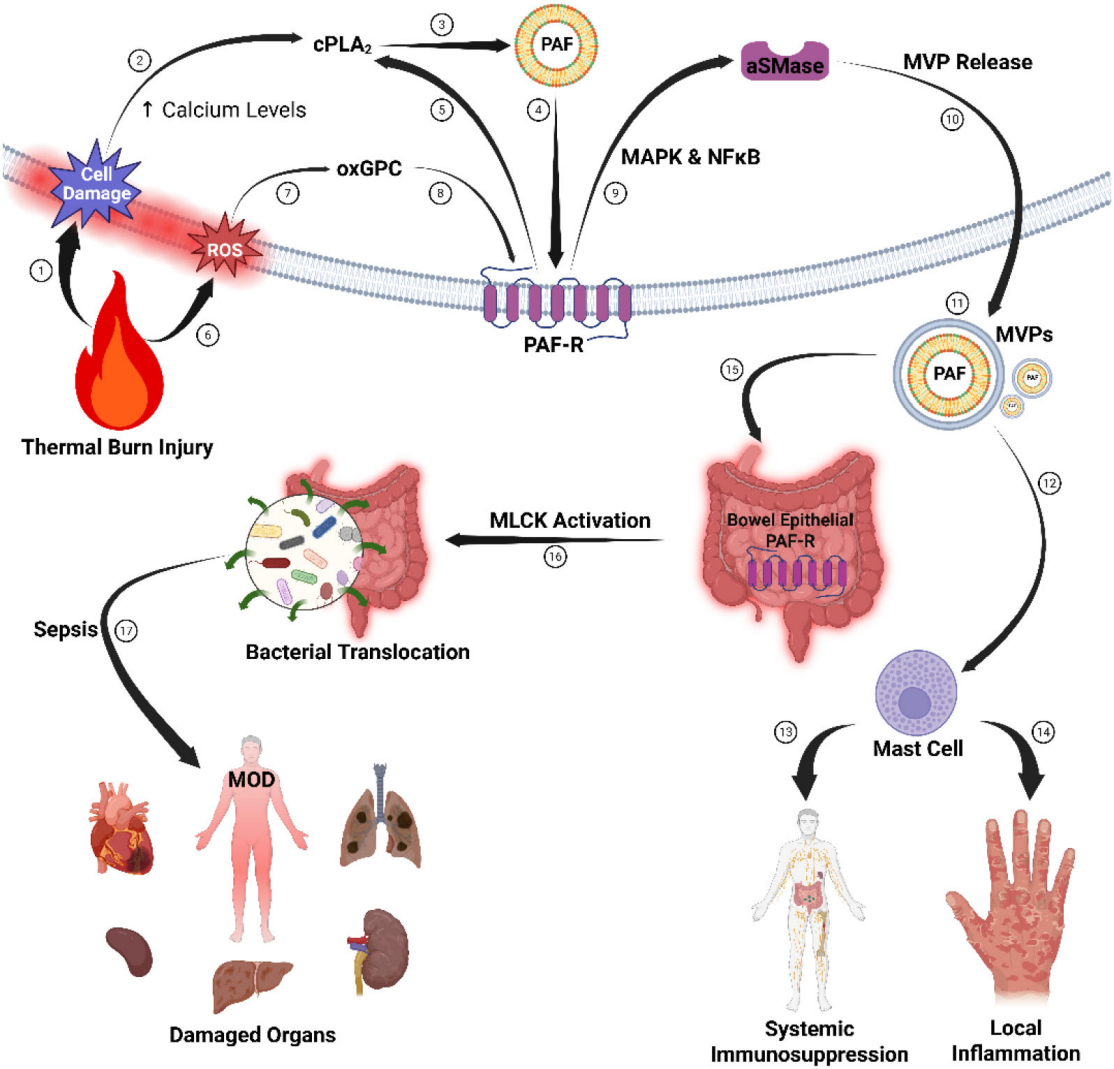
A conceptual pathway where thermal burn injury triggers the generation and release of MVPs carrying PAF agonists, which then could contribute to a range of physiological responses from localized inflammation to multiple organ dysfunction. This model depicts how (1) environmental stressors, such as a thermal burn injury, inflict cell damage on keratinocytes (2) causing increased intracellular calcium (Ca^2+^) levels (3) and this increased Ca^2+^ activates intracellular cytosolic phospholipase A_2_ (cPLA_2_), leading to the enzymatic generation of Platelet-activating Factor (PAF). (4) The enzymatic PAF activates and amplifies the Platelet-activating Factor receptor (PAFR) (5) activating additional cPLA_2_ and PAF biosynthesis, creating a positive feedback loop. (6) Environmental stressors also induce the formation of Reactive Oxygen Species (ROS) (7) which trigger the non-enzymatic production of oxidized glycerophosphocholines (oxGPC) (8) which activate the PAFR, contributing to further enzymatic PAF synthesis. (9) Activation of the PAFR also causes the enzyme acid sphingomyelinase (aSMase) to translocate to the plasma membrane through the mitogen-activated protein kinase (MAPK) pathway. (10) The translocated aSMase forms microvesicle particles (MVPs) and (11) these newly formed MVPs carrying PAF and oxGPC are released from the keratinocytes. (12) PAFR agonist-laden MVPs interact with PAFR-positive inflammatory cells, including mast cells (13) contributing to systemic immunosuppression as mast cells migrate to draining lymph nodes, promoting the formation of regulatory T cells (Treg), (14) also local inflammation, largely due to mast cell degranulation. (15) The MVPs can travel systemically to the intestinal epithelium and activate the bowel epithelial PAFR. (16) Stimulation of the intestinal epithelial PAFR leads to the activation of myosin light chain kinase (MLCK), which compromises the intestinal barrier. (17) The compromised intestinal barrier allows bacterial translocation from the gut, resulting in systemic bacteremia and sepsis. The widespread inflammation and concomitant sepsis lead to Multiple Organ Dysfunction (MOD).

Previous burn injury research has been complemented by our own group’s mechanistic murine studies which have focused on lipid PAF receptor signaling and subcellular microvesicle particles. Damage to the keratinocyte by heat or cold injury results in PAF biosynthesis (58). Our studies have also revealed that ethanol significantly augments the enzymatic production of PAF in skin keratinocytes during thermal burn injury through hyper-activation of the PAF-generating enzyme cytosolic phospholipase A_2_ (27, 59, 63). The PAF generated in response to TBI (with increased amounts in response to intoxicated TBI) then activate PAF receptors which then trigger the translocation of the enzyme acid sphingomyelinase (aSMase) to the plasma membrane which then generates MVP (28, 63–66). As outlined in **Figure 1**, these MVPs which carry the PAF agonists residing in the plasma membranes released from the skin keratinocyte act as critical mediators of systemic effects. It has been hypothesized that PAF is protected from degradation by travelling in MVPs throughout the body and thus allowing activation of PAF receptors in the gut, which in turn increases intestinal permeability and leads to bacterial translocation (27, 67). Additionally, PAF-laden MVP could also interact with the mast cell PAFR which will generate immunosuppression (65, 68), a known effect of TBI (26). Consistent with this hypothetical pathway, mice deficient in PAFR or aSMase lack increased systemic MVP, bacterial translocation and the increased multi-organ inflammation and systemic immunosuppression in response to intoxicated TBI (27, 28). This molecular mechanism provides a plausible explanation for the heightened systemic inflammation and gut barrier dysfunction and systemic immunosuppression observed in previously reported burn models. Collectively, these murine intoxicated TBI models not only clarify clinical observations of poorer outcomes in intoxicated burn patients but also identify MVPs as promising potential biomarkers and therapeutic targets, as evidenced by the attenuation of inflammation and systemic immunosuppression with a topical aSMase inhibitor applied immediately post-burn (27, 28).

In addition to MVP carrying PAF agonists, other potential carried bioactive agents have been associated with burn-derived MVP. In particular, the damage-associated High-Motility Group Box-1 (HMGB1) protein has also been found in TBI-derived MVP (69). Of importance, HMGB1-IL-1β complexes identified in the plasma from both murine and human TBI were found to be bioactive as they generated multiple proinflammatory cytokines (e.g., IL-6, IFN-γ) when added to a monocyte cell line (69).

### 3.3 Mechanisms of MVP release from skin

A variety of events, such as oxidative stress, heat damage, inflammatory cytokines, and physical trauma, cause skin cells to release MVP (67). *In vitro* studies have documented that MVPs can carry PAF and in a physiologically protective vesicular form in response to TBI (63, 70, 71). Enzymatic PAF synthesis can be triggered by a range of stimuli, including ultraviolet B radiation (UVB), thermal, and cold injury (57, 58, 70). MVP biogenesis is significantly increased by thermal burn damage, particularly when it is coupled with acute ethanol intoxication (intoxicated TBI, or ITBI). The generation and release of MVP in response to multiple stressors can involve PAFR signaling (67).

In this model, TBI induce MVP release and cause both systemic immunosuppression and damage to multiple systemic organs via complex molecular pathways involving activation of aSMase, the myosin light-chain kinase (MLCK) pathway, and PAF signaling (71). Detailed in **Figure 1**, PAF production is triggered when thermal stress activates cytosolic phospholipase A_2_ (cPLA_2_) (27, 59). The enzyme aSMase is activated when PAF binds to the PAFR on keratinocytes, which sets off downstream signaling through mitogen-activated protein kinase (MAPK) and nuclear factor kappa B (NF-κB) (61). The PAF-PAFR axis is essential because, in both cellular keratinocyte and murine models, pharmacologic suppression of cytoplasmic phospholipase A_2_ (cPLA_2_) or PAFR or genetic KO of the PAFR prevents MVP release (27, 61, 63). Ceramide produced by aSMase activation encourages membrane curvature and budding, which appear to be essential for MVP production and release (61). Systemic immunosuppression would be triggered when MVPs transport PAF to mast cells, activating PAFR and encouraging mast cell migration to lymph nodes where they increase regulatory T cells as well as immunosuppressive cytokines including IL-10 and TGF-β (28, 61, 65, 68). PAF-enriched MVP attach themselves to the intestinal epithelium PAFR, causing actomyosin contraction, MLCK activation, increased gut permeability and epithelial permeability. This permits bacteria and endotoxin to translocate into the host, resulting in coagulation activation, endotoxemia, inflammatory cytokine storms, and MOD (27).

As previous research has shown, the mortality rate within the elderly population is much higher following extensive thermal burn injuries, citing complications from the weakened immune response that can be mounted (72). Studies with animal models has recently shown that cutaneous burns in aged animals induce increased gut permeability, further causing infiltration of microbiome bacteria and toxins in comparison to young mice (40). We propose that PAF traveling via MVP released from the burn site could also serve as the effectors in the geriatric population.

### 3.4 Influence of ethanol and aging on TBI outcomes

The presence of ethanol in the blood of burned victims, as previously demonstrated in animal models, exacerbates the MVP release from burn injuries leading to diminished immune responses and increased gut permeability. In humans, burn victims who have concomitant ethanol intoxication have poorer outcomes (15, 34, 73, 74). The pathology in humans appears to mimic the murine model systems with increased cytokine and neutrophil activation systemically, resulting in end organ damage being more dramatic as compared to non-ethanol exposed subjects. In our proposed model (**Figure 1**), increased enzymatic PAF generation by the combination of ethanol and TBI would explain the increased pathologies.

Older people are more vulnerable to TBI systemic effects due to senescent immunological changes, which include altered cellular signaling pathways, reduced innate and adaptive immune responses, and chronic inflammation (inflammaging) (75). Thinner epidermis and dermis, decreased vascularity, decreased keratinocyte turnover, and compromised barrier integrity are all signs of morphological and functional degeneration in aged skin, which makes deeper burns and slower healing more likely (76, 77).

Comorbid disorders including cardiovascular disease, diabetes, and overall increased frailty are common in elderly burn patients, which reduces resilience and makes resuscitation, wound care, and rehabilitation more difficult. Elderly burn patients are more susceptible to sepsis and MOD, both major contributors to post-burn mortality (74). The amplified systemic inflammatory response, combined with impaired immune resolution mechanisms, promotes systemic cytokine release and vascular dysfunction (40).

### 3.5 Evidence that TBI generates systemic MVP in humans

**Figure 2:**
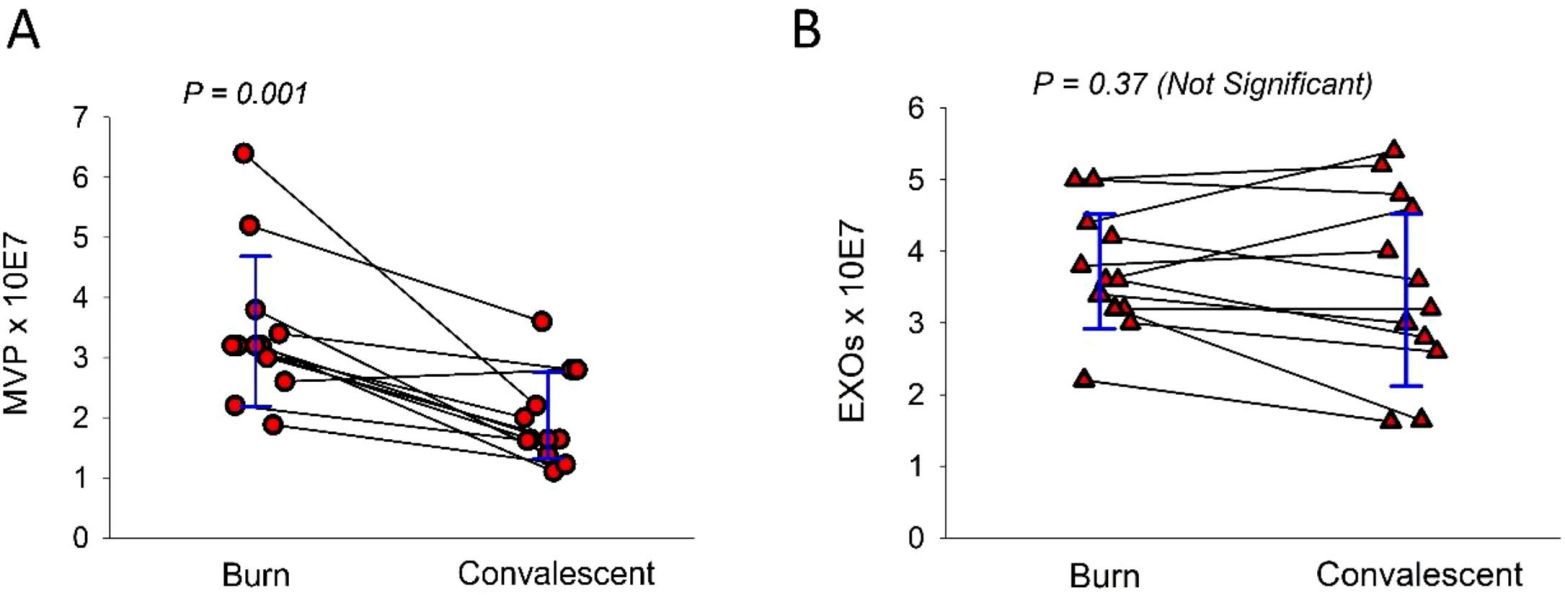
Pilot studies suggesting that levels of subcellular MVP but not exosomes are acutely elevated in the plasma following a TBI. 12 subjects were recruited to this study in which 10 mL of blood was acquired within 12 hours of a significant TBI and 24–116 days later a convalescent blood sample was obtained. The levels of MVP **(A)** and exosomes (Exos) **(B)** were quantified and expressed per microliter of plasma. The data represent paired burn and convalescent samples for the 12 subjects. Error bars are +/- SD. Statistical analysis used a paired two-sample t-test.

Though both *in vitro* cellular and *ex vivo* human skin and preclinical *in vivo* murine studies have demonstrated that TBI is a potent stimulus for MVP release (27, 70), it is unknown whether humans suffering an acute TBI generate systemic MVP. To that end, we initiated pilot studies in which patients who were admitted to the Premier Medical Center Burn Unit (Dayton, Ohio, USA) within 12 hours of a thermal burn injury were enrolled. These studies involving volunteer adult humans were approved by the Wright State University Institutional Review Board, Dayton Ohio (IRB-2023-422) and followed the Declaration of Helsinki Principles. In these studies, following informed consent, 10 mL of whole blood (collected in heparinized tubes) was obtained from the subject at admission to the Burn Unit and a second 10 mL sample obtained at convalescence (24–116 days later). Blood was spun to separate plasma and the plasma underwent differential centrifugation as per our previously published methodologies (61, 70) to obtain MVP and smaller exosomes (small extracellular vesicles). As shown in **Figure 2**, 12 subjects were tested (4 females; 8 males, aged 23-64; estimated body surface areas ranging from approximately 3-50%) which revealed statistically significant elevated levels of MVPs in the initial sample taken around the time of the initial TBI (within 12 hours) in comparison to the convalescent values. Of interest, the levels of exosomes in this population were unchanged (**Figure 2B**) suggesting that the elevated MVP levels at the initial sampling was likely not due to concentration differences attributable to changes in fluid status. Though these exploratory pilot studies have significant limitations including small sample size (n = 12) and clinical heterogeneity, they do provide some support for a possible role of MVP in acute human TBI. Importantly, the continuation and expansion of studies such as this should allow this hypothesis to be tested in humans.

## 4 Current management of TBI systemic effects

### 4.1 Acute care

Acutely, systemic effects of thermal burn injuries are met with the ABCs of emergency medicine, circulation, airway and breathing. This primary survey occurs prior to more invasive maintenance measures. To address the most immediate complications of hypovolemia and hypothermia, intravenous fluids are provided to prevent and treat shock or the diminished perfusion and oxygen delivery to end-organs. Monitoring urine output as well as other end points of resuscitation are helpful gauges to determine resuscitation status. Wound care, antimicrobials, and fighting oxidative stress through electrolyte supplementation are also current measures often utilized (78).

Systemically, inhalation injuries, extensive TBSA burns, and patients with pre-existing comorbidities that impede healing, or impede circulation will need more intensive care. A Baux score can be calculated which can determine the risk of death as a complication, and other predictive measures can be utilized to determine blanket mortality risk, duration of hospital stay, or the necessity for ICU intervention (16, 79).

### 4.2 Chronic care

Preventing dehydration, pain medications, creams and ointments to keep the burn sites moist, antibiotics for infections are all acceptable treatment strategies. Chronically, burn injuries take an extensive amount of time to heal and patients must be diligent in management to regain their original function. Physical therapy, pharmacological pain treatment, and outpatient management with diligent wound care is a common regimen pursued. Long-term management of TBI often involves a multi-disciplinary approach including psychological rehabilitation and coping with the offending trauma, establishing a dietary caloric minimum to compensate for the hypermetabolic state and recovery, as well as regular follow-up with wound care professionals for burn management and to avoid complications including skin contractures, sepsis, keloid formation, and chronic hypermetabolism. Additionally, care to manage the long-term sequelae of the MOD including but not limited to renal, hepatic, cardiac, pulmonary, neurologic, and gastrointestinal failure is needed.

### 4.3 Need for improved management

Though management of the burn victim has been improved via specialized care in dedicated burn centers (7), the standard acute treatments have not changed appreciably in the last several decades. Thus, there is a compelling need for further research in TBI. An improved understanding of the acute TBI pathophysiology should result in new pharmacologic targets to address the potentially lethal MOD as well as the increased susceptibility to infections associated with the acquired immune deficits in the setting of barrier disruption.

### 4.4 Therapeutic implications & future research opportunities

Acute MOD as discussed is a critical and mortality predicting complication of TBI that is currently managed through ICU level fluid resuscitation and hemodynamic monitoring. However, there is no evidence that fluid monitoring and replacement alone could interrupt the enzymatic cascades resulting in the release of MVPs.

Therapeutic interventions that have recently been explored in animal models to combat MOD and further complications from TBI include MLCK inhibitors (38) and anti-IL-6 antibodies (38). Based upon our model, there is premise for the testing of FIASMs (functional inhibitors of acid-sphingomyelinase) to control MVP release as well as regulate cell signaling (80). Beyond the aSMases and their role within the ceramide cascade that is being researched regarding viral cell signaling (81), this enzyme as previously discussed plays an essential role in the release of MVPs containing PAF following TBI. Utilizing FIASMs including tricyclic antidepressants such as amitriptyline and imipramine which have been shown in prior animal models to inhibit the MVP release following TBI could be repurposed for human studies (27, 28). We have recently demonstrated that topical treatment with imipramine immediately following localized UVB irradiation blocks MVP release in skin (82). An advantage of this approach targeting aSMase is that it could also potentially inhibit the formation of the release of other putative bioactive MVP-carried agents such as HMGB1-IL-1β complexes (69). Further research in this realm is needed to assess efficacy of these relatively safe agents in acute human burn patients.

Other avenues to be explored regarding this area of research include the targeting of multiple enzymatic components of the established cascade, such as the addition of an MLCK inhibitor, FIASM, and/or introducing a “triple therapy” that also involves inhibition of PAF receptors. At present there is only one PAFR antagonist (rupatadine) which is commercially available (83). It should be noted that the vast majority of PAFR antagonists are far less potent than the native 1-alkyl PAF ligand (84). These therapeutic tools in humans may provide evidence for this pathway involving PAF-laden MVPs as well as could potentially revolutionize initial management of the acute TBI patient.

Finally, more clinically-oriented studies similar to the pilot studies presented in this manuscript should be initiated. Data from large numbers of acute TBI patients will allow comparisons of MVP levels with clinical parameters that are known to result in worse outcomes to include age, % body surface area of burn, and presence of ethanol intoxication.

## 5 Conclusions

The PAFR-aSMase-MVP axis could potentially serve as a critical mechanism which could provide an explanation for how localized cutaneous damage can escalate into severe systemic pathologies. Compelling data from murine studies provide support for MVP involvement in acute TBI-related pathologies. The exploratory human studies presented in this manuscript also fit with the notion that burn-derived MVP could serve as important contributors to the pathologies associated with severe TBI. An improved understanding of the role that MVPs that carry PAF and potentially other mediators play in mediating systemic reactions to burns, such as vascular dysfunction, immunological dysregulation, and increased gut permeability, is crucial. The present-day treatments mainly comprise wound care, infection control, and fluid resuscitation as supportive treatments that do not directly address the upstream signaling cascades which eventually translate into systemic toxicity. Future pharmaceutical approaches may provide novel means for prophylaxis or reduction of systemic complications by targeting molecular mediators such as PAF and aSMase-dependent MVP release identified to play roles in pathophysiology. The success of pharmacological aSMase inhibitors, for example imipramine, in attenuating ITBI-induced systemic inflammation and MVP release in mice, underscore the immense therapeutic potential of targeting this pathway. This research could change how acute TBI is managed, aiming at the molecular mechanisms of the disease to decrease mortality and improve long-term outcomes.
